# Successful autologous stem cell transplantation for light chain proximal tubulopathy with severe kidney injury

**DOI:** 10.1002/ccr3.8337

**Published:** 2023-12-13

**Authors:** Asuka Kono, Kana Bando, Atsushi Takahata, Shigeo Toyota

**Affiliations:** ^1^ Department of Hematology Yokosuka Kyosai Hospital Yokosuka Japan

**Keywords:** Fanconi syndrome, hematopoietic stem cell transplantation, monoclonal Gammopathy of undetermined significance, multiple myeloma, proximal renal tubular dysfunction

## Abstract

Light chain proximal tubulopathy (LCPT) is a rare type of monoclonal gammopathy of renal significance. Clinicians should consider LCPT in the differential diagnosis of patients with renal or proximal tubular dysfunction with monoclonal gammopathy. They should confirm diagnosis by renal biopsy and initiate chemotherapy before disease progression.

## Graphical abstract

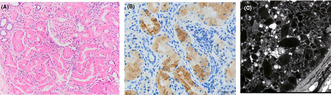

## 1 INTRODUCTION

Monoclonal gammopathy is associated with various types of renal injuries, such as light chain cast nephropathy, amyloid light chain (AL) amyloidosis, and monoclonal immunoglobulin deposition disease, recently recognized as monoclonal gammopathy of renal significance (MGRS). Light chain proximal tubulopathy (LCPT) is a rare type of MGRS, with approximately only 150–200 cases reported in the literature.^1^, ^2^, ^3^, ^4^, ^5^, ^6^ In LCPT, monoclonal light chains secreted by abnormal plasma cells accumulate in the proximal tubular cells and cause proximal tubular dysfunction, which is clinically characterized by tubular acidosis, normoglycemic glycosuria, aminoaciduria, and hypophosphatemia, collectively called Fanconi syndrome.^7^

Diagnosis of LCPT is confirmed by renal biopsy findings and the presence of specific histological features, including cytoplasmic monoclonal light chain inclusions and an increased number of lysosomes in the proximal tubular cells, which are sometimes only detectable by electron microscopy.^8^ Additionally, the coexistence of LCPT and other paraprotein‐related kidney diseases has been reported,^9^, ^10^, ^11^ which makes the diagnosis more difficult. LCPT often presents as slowly progressive renal impairment; however, some patients develop end‐stage kidney disease or aggressive multiple myeloma.^7^

Although the treatment strategy for MGRS has not yet been established due to the rarity of and lack of familiarity with this entity, several studies have shown that improvement in renal function can be achieved with hematologic response to chemotherapy, commonly bortezomib for plasma cell dyscrasia and rituximab for B cell lymphoproliferative disease. Some case reports and case series have shown that chemotherapy directed at multiple myeloma is also effective for LCPT,^1^, ^2^, ^3^, ^4^ but only a few case reports have described its clinical course, and the optimal treatment strategy remains unknown.

Herein, we describe the case of a patient with LCPT and severe kidney injury who received bortezomib‐based chemotherapy and autologous stem cell transplantation (ASCT). This case report provides new insight into the optimal treatment strategy for LCPT in the future.

## 2 CASE REPORT

A 64‐year‐old Japanese man with a history of hypertension, gastric cancer, and mild thrombocytopenia had renal dysfunction (creatinine, 1.5 mg/dL) and hypokalemia 3 years prior and was referred to our nephrologist because of progressive renal impairment and proximal tubular dysfunction. His laboratory data are shown in Table 1.

**TABLE 1:** Laboratory findings at diagnosis.

Urinalysis		Complete blood count		Immunology	
pH	5.5	WBC	6800 μL	IgG	1869 mg/dL
Protein	2+	Hb	13.8 g/dL	IgA	131 mg/dL
Blood	1+	Plt	130,000 /μL	IgM	65.4 mg/dL
Glucose	2+	Biochemistry		IgG4	8.9 mg/dL
U‐TP	1.33 g/day	TP	7.9 g/dL	ACE	9.6 U/L
U‐Cr	0.96 g/day	Alb	4.3 g/dL	SS‐A	<0.5 U/mL
U‐K	42.2 mEq/day	γ‐fraction	24.9%	SS‐B	<0.5 U/mL
U‐P	396 mg/dL	BUN	24 mg/dL	amyloid A	7 mg/dL
U‐UA	383 mg/day	Cr	2.91 mg/dL	β2MG	5.2 mg/L
U‐NAG	23.1 U/L	UA	1.9 mg/dL		
U‐β2MG	82,561 μg/dL	Na	140 mEq/L	IFE	
FEUA	50%	K	4.4 mEq/L	Of urine	FLC‐κ
FEK	37.5%	Cl	111 mEq/L	Of serum	IgG‐κ
%TRP	59%	Ca	9.2 mg/dL	FLC‐κ	567 mg/L
Vein Blood Gas		P	3 mg/dL	FLC‐λ	33 mg/L
pH	7.271	AST	13 U/L		
HCO3‐	21.2	ALT	15 U/L		
BE	−5.4	T‐bil	0.5 mg/dL		

Abbreviations: ACE, angiotensin converting enzyme; Alb, albumin; ALT, alanine transaminase; AST, aspartate aminotransferase; β2MG, β2 microglobulin; BE, basic excess; BUN, blood urea nitrogen; Cr, creatinine; FEK, fractional excretion of potassium; FEUA, fractional excretion of uric acid; FLC, free light chain; Hb, hemoglobin; IFE, immunofixation electrophoresis; Ig, immunoglobulin; NA, not available; NAG, N‐acetyl‐β‐D‐glucosaminidase; Plt, platelets; pro, protein; SS, Sjögren's syndrome; T‐bil, total bilirubin; TP, total protein; TRP, tubular reabsorption of phosphate; U, urinary; UA, uric acid; WBC, white blood cell; γ‐fraction, gamma fraction of serum globulin.

A 24‐h urine collection test revealed massive proteinuria (2+, 1.33 g/day) without hematuria, and the test results for the Bence–Jones protein were positive. The urinary beta‐2 microglobulin level was markedly elevated to 92,279 ng/mL. The serum potassium and uric acid levels were 4.4 mEq/L (under oral potassium supplementation) and 1.9 mg/dL, respectively. The serum creatinine level was 2.91 mg/dL (estimated glomerular filtration rate [eGFR], 18.7 mL/min/1.73m^2^). Serum protein electrophoresis showed an M spike (1.82 g/dL). The κ free light chain level was elevated to 56.7 mg/dL. Regarding the renal tubular function, the fractional excretion of uric acid (FEUA; normal range, 4%–14%) and potassium (normal range, 10%–20%) were 50% and 37.5%, respectively, and the tubular reabsorption of phosphate (TURP; normal range, 60%–90%) was 59%.

A bone marrow examination showed normocellular marrow with 5% plasma cells exhibiting κ light chain clonality. Cytogenic analysis using the G‐banding technique showed a normal karyotype, and metaphase fluorescence in‐situ hybridization revealed a 1 q gain of 3% (three copies) without any other high‐risk chromosomal abnormalities, including t(4;14)(p16;q32), t(14;16)(q32;q23), or deletion(17p).

**FIGURE 1:**
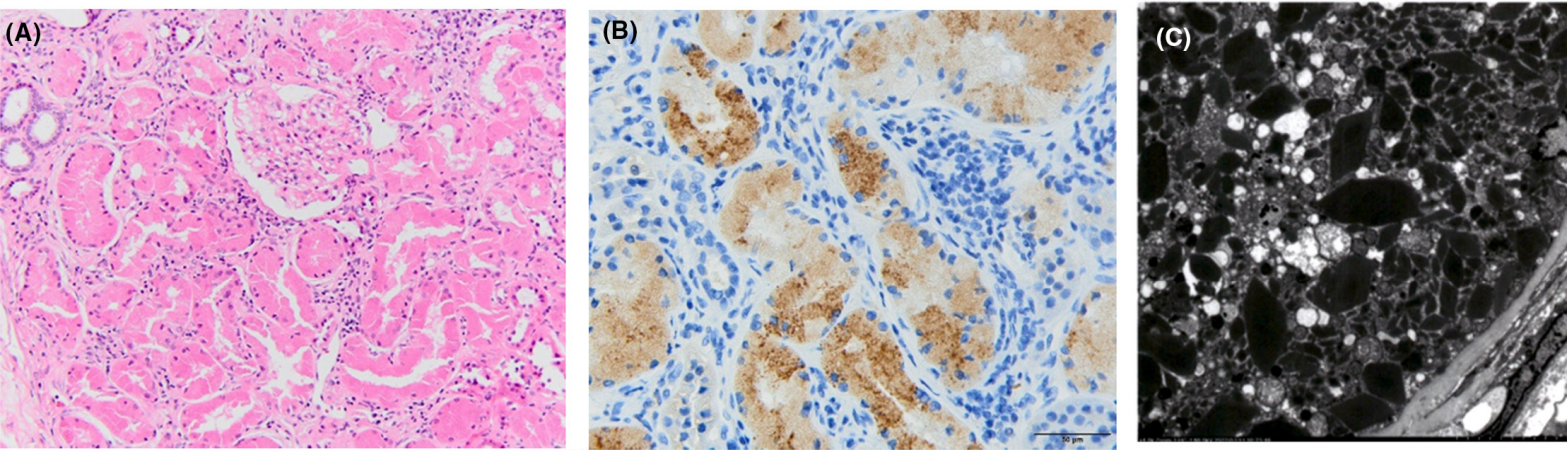
Renal biopsy findings. Light microscopic photomicrographs of glomeruli and proximal tubules. Hematoxylin and eosin staining image (A, ×200) shows no remarkable abnormalities. Immunofluorescence staining image of kappa light chain in proximal tubular epithelial cells (B, ×400). Electron microscopic image of proximal tubules (C) shows rhomboid crystal deposition in the proximal tubular epithelial cells.

Renal biopsy and immunofluorescence analysis revealed that none of the glomeruli showed histological abnormalities, including amyloid or immunocomplex deposition. However, the κ light chain was positive in the proximal renal tubules, and electron microscopy revealed light chain proximal tubulopathy‐specific crystals in the proximal tubular epithelial cells (Figure 1). A [^18^F]Fluorodeoxyglucose positron emission tomography‐computed tomography scan revealed no additional renal signs of light chain deposition disease or bone lesions suggestive of multiple myeloma.

**FIGURE 2:**
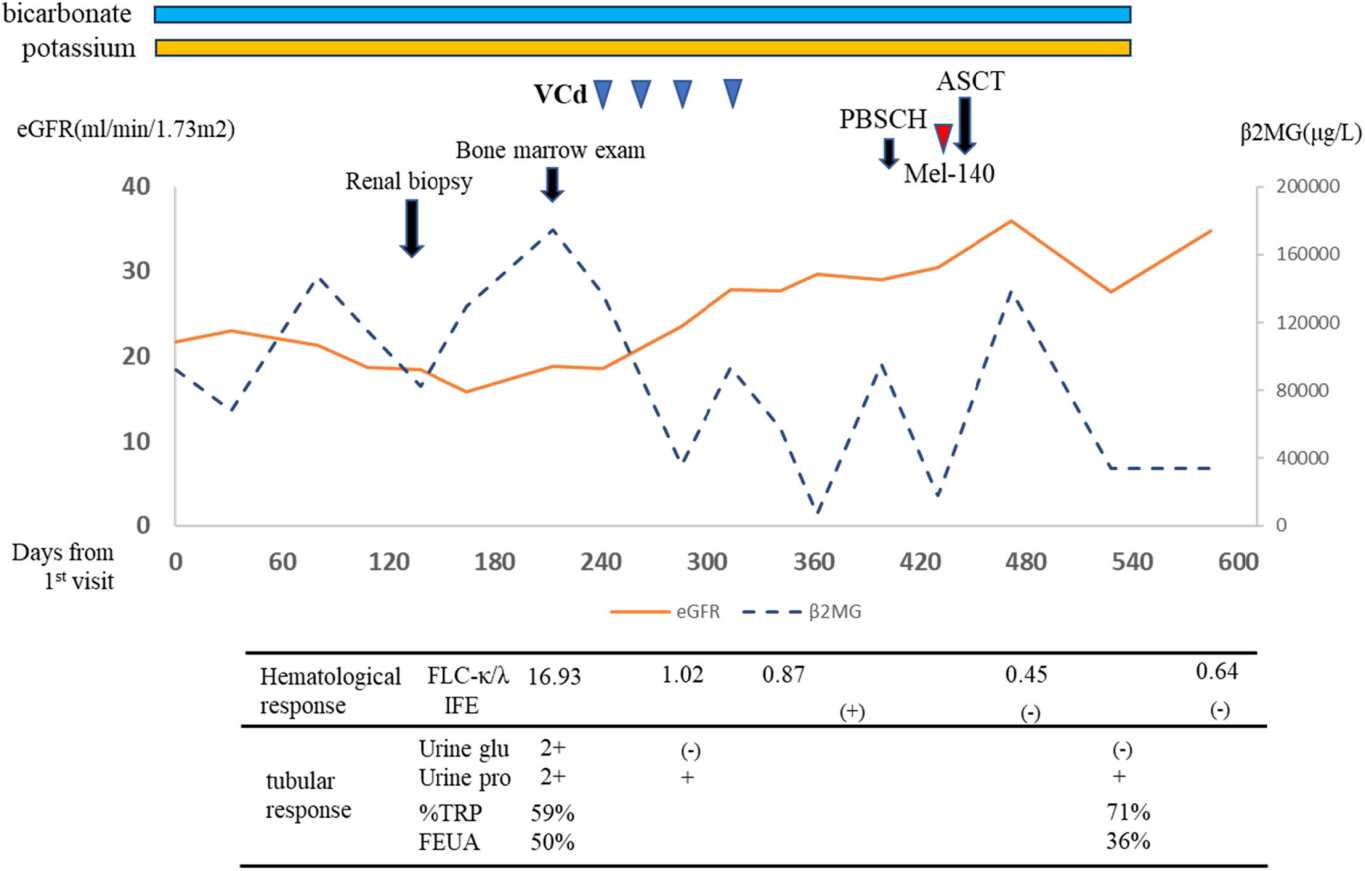
Clinical course of the patient. ASCT, autologous stem cell transplantation; eGFR; estimated glomerular filtration rate; FEUA, fractional excretion of uric acid; FLC, free light chain; Mel‐140, high‐dose melphalan (140 mg/m^2^); PBSCH, peripheral blood stem cell harvest; TRP, tubular reabsorption of phosphate; UCr, urine creatinine; UP, urine protein; VCd, bortezomib, cyclophosphamide, and dexamethasone; β2MG, β2‐microglobulin.

The clinical course of the patient is shown in Figure 2. Following an established diagnosis of LCPT with monoclonal gammopathy of undetermined significance, bortezomib 1.5 mg/m^2^, cyclophosphamide 300 mg/m^2^, and dexamethasone 40 mg were administered on Days 1, 8, 15, and 22. After four treatment cycles, serum κ light chain and creatinine improved to 24.9 mg/L (pretreatment, 567 mg/L) and 1.87 mg/dL, respectively. Improvements in the renal function prompted us to initiate ASCT, and the patient underwent peripheral blood stem cell mobilization with granulocyte colony‐stimulating factor and plerixafor. Subsequently, he received a high dose of melphalan (140 mg/m^2^) followed by ASCT and achieved successful engraftment without serious complications.

One month after ASCT, the patient achieved a stringent complete response, and the serum creatinine level decreased from 3.32 to 1.85 mg/dL. In addition, proximal tubular function improved, as evidenced by a reduction in FEUA, elevation of TURP, and resolution of urinary glucose. The patient no longer needed potassium or bicarbonate supplementation. His renal function remained stable for 9 months after ASCT.

## 3 DISCUSSION

LCPT is a rare subtype of MGRS, and the treatment strategy remains controversial, as well as the other types. Previous studies have not supported the use of chemotherapy drugs, such as alkylating agents, because of their ineffectiveness in improving renal function and adverse effects, such as secondary malignancy.^5^ However, as new drugs for multiple myeloma have become available, 11 case reports and 4 case series have shown the efficacy of chemotherapy, particularly bortezomib‐based regimens and ASCT to improve renal and tubular function^12^, ^13^, ^14^, ^15^, ^16^, ^17^, ^18^, ^19^, ^20^, ^21^, ^22^ (summarized in Tables 2 and 3). Although these studies comprised a small case series and the definition of the renal outcome was variable, all reports showed that chemotherapy improved renal function or delayed the progression to end‐stage kidney disease, compared with that in the non‐chemotherapy group (Table 2). In addition, they reported an improvement in renal tubular function, characterized by the resolution of proteinuria or urinary glucose and the elevation of serum phosphate and uric acid levels (Table 2).

**TABLE 2:** Reported outcomes of chemotherapy for LCPT.

Reference	*n*	Age (years)	Underlying disease	Chemotherapy	Hematological response	Renal response	Baseline kidney function	Renal outcome (chemo vs. non‐chemo)	Follow‐up (mean)
^1^	46	62.5 (39–87)	MM 15 sMM 7 MGUS 21 other 3	Total 27/36 Bor 12 IMiDs 15 ASCT 11	CR 5/22 VGPR 1/22 PR 2/22 SD 14/22	Improve 7/22 Stable 11/22 Worsening 4/22 ESKD 0/22	Median eGFR Chemo: 38.5 Non‐chemo: 34.8	ESKD 0/22 vs. 2/8 ΔeGFR 5.1 vs. ‐6.9	39 months (1–141)
^2^	49	58 (37–84)	MM 11 sMM 25 MGUS 13	Total 42/49 Bor 11 IMiDs 7 Alkylating 6 ASCT 14	CR 4/34 VGPR 10/34 PR 18/34 SD 2/34	Improve/stable 34/38 ESKD 9/38 Tubular response 2/38	Median eGFR 33 (4–111)	ESKD 5/41 vs. 4/8	3.8 years (1–25)
^3^	22	49 (30–76)	MM 6 sMM 2 MGUS 13 other 1	Total 12/22 Bor 7 IMiDs 9 ASCT 2	CR 3/18 VGPR 2/18 PR 6/18 SD 3/18	Improve 4/9 ESKD ND Tubular response10/15	Median eGFR Chemo: 80 Non‐chemo: 60	ΔeGFR% 12.1% vs. −8.9%	ND
^4^	26	54.7 (40–69)	MM 10 sMM NA MGUS 14 other 2	Total 13/26 Bor 6 IMiDs 10 Mel 2 ASCT 1	CR 3/12 VGPR /PR 6/12 SD/PD 3/12	Improve/ Stable 7/12 ESKD 0/12 Tubular response 8/12	Mean eGFR 68.0 ± 26.4	eGFR at 6 months 80.6 vs. 61.8	36 months (0–133)

Abbreviations: ASCT, autologous stem cell transplantation; Bor, bortezomib; CR, complete response; eGFR, estimated glomerular filtration rate; ESKD, end stage kidney disease; IMiDs, lenalidomide or thalidomide; Mel, melphalan; MGUS, monoclonal gammopathy of undetermined significance; MM, multiple myeloma; PD, progressive disease; PR, partial response; SD, stable disease; sMM, smoldering multiple myeloma; VGPR, very good partial response.

**TABLE 3:** Case reports of chemotherapy for LCPT.

Reference	Age/sex	Underlying disease	Histology	Chemotherapy	Hematological response	Renal response	Tubular response	Follow‐up
12	50s/F	Low grade BCL (IgM‐κ)	κ + PT	Benda+RTX	ND	sCr 1.45 → 1.1	Urine β2MG, UA	1 year
13	76/M	WM (IgM‐κ)	Crystal in PT	Vd + RTX	Response	ND	No response	Death
14	45/F	MM (FLC‐κ)	κ + crystals in PT	VTd (4)	CR	sCr 2.6 → normal	ND	ND
15	59/M	MM (IgA‐κ)	Crystals in PT, κ	VCd (5), ASCT	CR	sCr 2.0 → 1.4	Urine protein	3 years
16	47/F	MUGS (κ)	Crystal in PT	Vd (3), ASCT	CR	sCr 2.0 → 1.52–1.87	Urine protein	2 years
17	67/M	MGUS (IgG‐κ)	Increased lysosomes κ + PT, no crystalline	Vd	SD	Stable	No tubular injury	ND
Our case	64/M	MGUS (IgG‐κ)	κ + crystals in PT	VCd (4), ASCT	sCR	sCr 3.03 → 1.9 eGFR15.8 → 34.8	Urine protein, glucose, UA, K, P	1 year
18	45/M	MGUS (FLC‐λ)	No deposit	VCd	sCR	eGFR 45 → 84	Urine protein, glucose	3 months
19	70/M	MGUS (IgG‐λ)	λ + PT	Vd	≧PR	sCr 3.91 → 1.35	Urine protein	7 months
20	73/F	MM (IgG‐λ)	λ + PT	Rd → IRd	NA	eGFR 47 → 55	Urine β2MG	1 year
21	77/M	MM (IgD‐λ)	λ + PT, no crystalline	V^a^	CR	eGFR 56.7 → 85	No tubular injury	ND
22	78/F	MM (IgD‐λ)	λ + droplet, no crystalline	Vd	VGPR	eGFR 14.3 → 15	Urine protein, β2MG	ND

Abbreviations: ASCT, autologous stem cell transplantation; BCL, B‐cell lymphoma; Benda, Bendamustine; C, cyclophosphamide; CR, complete response; d, dexamethasone; eGFR, estimated glomerular filtration rate; ESRD, end stage renal disease; FLC, free light chain; I, ixazomib; IG, immunoglobulin; MGUS, monoclonal gammopathy of undetermined significance; MM, multiple myeloma; ND, not described; PR, partial response; PT, proximal tubules; R, lenalidomide; RTX, Rituximab; sCr, serum creatinine; sMM, smoldering multiple myeloma; T, thalidomide; UA, uric acid; V, bortezomib; VGPR, very good partial response; β2MG, β2‐microglobulin; WM, Waldenstrom macroglobulinemia.

^a^ Bortezomib including regimen.

Although we cannot precisely assess the efficacy of different treatments owing to the small size and heterogeneity of the series, a combination of chemotherapy and ASCT tended to achieve a better renal or proximal tubular function than chemotherapy alone.^1^, ^2^ These data suggest that renal and tubular functional improvements can be achieved by reducing the secretion of free light chains by abnormal plasma cells. Although emerging evidence suggests that the organ response rate may be improved by negative minimal residual disease in AL amyloidosis, the most common type of MGRS,^23^ no data are available on whether the complete eradication of abnormal plasma cells is beneficial for the kidneys or delays the progression to multiple myeloma in patients with LCPT.

Recently, daratumumab, an anti‐cluster of differentiation (CD)38 monoclonal antibody, has been commonly administered for multiple myeloma and AL amyloidosis. As most of the case reports were published before the approval of daratumumab, the administration of daratumumab for LCPT was limited to only two cases, complicated with AL amyloidosis and crystal‐storing histiocytosis, respectively.^9^, ^11^ Both were efficacious and the CD38 monoclonal antibody seems to be a promising therapeutic alternative for LCPT.

It is noteworthy that the renal function improved in our case despite the severe initial kidney injury in comparison to the extent of renal injury in the previous case reports (Table 3). Although most previous reports include eGFR above 30 mL/min/1.73m^2^ or creatinine below 2.0 mg/dL, renal injury progressed to eGFR 15.4 mL/min/1.73m^2^ (creatinine, 3.31 mg/dL) at the initiation of treatment in our case. We found only two cases of LCPT with severe kidney injury, one of which showed no improvement of renal function,^22^ and the other which showed remarkable improvement, as observed in our case.^19^ These data imply that we should aggressively consider diagnosis by renal biopsy and chemotherapy for LCPT, even in cases with severe kidney impairment.

Our study limitation was the unclear beneficial effect of the addition of ASCT on clinical outcomes. According to our research, ASCT was performed in a total of only 28 cases in the four case series (Table 2)^1^, ^2^, ^3^, ^4^ and two case reports (Table 3)^15^, ^16^ and the role of ASCT in the treatment of LCPT cannot be precisely determined. We decided to perform ASCT considering that the patient responded well to chemotherapy with rapid improvement in renal function.

In conclusion, a combination of bortezomib‐based chemotherapy and ASCT could effectively treat LCPT and successfully improve the renal function in this patient. Therefore, hematologists should be familiar with LCPT as a differential diagnosis for renal impairment with monoclonal gammopathy of undetermined significance and multiple myeloma and should consider chemotherapy even with severe renal injury. However, data on the best treatment strategy and long‐term prognosis are still lacking. Larger prospective studies are needed to support our results and determine the optimal treatment strategy for LCPT.

## AUTHOR CONTRIBUTIONS

**Asuka Kono:** Data curation; investigation; methodology; writing – original draft; writing – review and editing. **Kana Bando:** Conceptualization. **Atsushi Takahata:** Project administration. **Shigeo Toyota:** Writing – review and editing.

## FUNDING INFORMATION

No finding was received for the preparation of this manuscript.

## CONFLICT OF INTEREST STATEMENT

None of the authors have any relevant conflicts of interest to declare.

## CONSENT STATEMENT

Written informed consent was obtained from the patient for publication of this case report and accompanying images.

## Data Availability

The data that support the findings of this study are available from the corresponding author upon reasonable request.
